# Distal Femur Cemented Modular Prosthesis Failure Causing a Medical Device-Related Adverse Event: A Report of a Rare Case

**DOI:** 10.7759/cureus.64823

**Published:** 2024-07-18

**Authors:** Arohi Agarwal, Krishna Singh, Samyak Jain, Gagan Singh, Puneet Dhamija, Piyush Mittal

**Affiliations:** 1 Materiovigilance, All India Institute of Medical Sciences Rishikesh, Rishikesh, IND; 2 Pharmacy Practice, Teerthanker Mahaveer College of Pharmacy, Moradabad, IND; 3 Materiovigilance, Indian Pharmacopoeia Commission, Ministry of Health and Family Welfare, Ghaziabad, IND; 4 Pharmacology, All India Institute of Medical Sciences Rishikesh, Rishikesh, IND

**Keywords:** megaprosthesis, osteoblast, distal femur prosthesis, mesenchymal cells, osteosarcoma

## Abstract

A distal femoral cemented modular prosthesis is a viable option for post-bone tumor and limb salvage procedures. The major reasons for implant failures are the poor quality of implants, mechanical stress, biochemical reactions, and extended period of the implant in vivo use. Rare incidences have been reported of distal femur prosthesis implant malfunctioning in a subject having osteosarcoma. Common adverse events associated with implant failure include surgical site infections, swelling, pain, revision of the surgical procedure, cyst formation, and build-up of metal debris on soft tissues. Our case report summarizes gross malfunctioning of a distal femur cemented modular prosthesis experienced by a 24-year-old post-operated osteosarcoma patient who developed excruciating sudden pain and the inability to bear weight on the right leg, with the sudden onset of these symptoms developing while turning in bed.

## Introduction

Osteosarcoma is a de novo disease process of the bone, accounting for more than 85% of cases globally, and is a type of bone-forming malignant tumor as classified by the World Health Organization. It is the second most common primary bone tumor with high malignant tendencies generally found in the second decade of life when there is a significantly high turnover of bones [1,2]. Osteosarcoma is derived from the mesenchymal cells in the bone marrow which produce osteoid substances and/or immature bone [3,4]. Mutations in the TP53 and RB1 genes of osteoblastic cells, or their antecedents, in the lower limbs, like the tibia or femur, cause tumorigenesis [5].

The most preferred and suitable choice for an orthopedic to correct bone defects in the femur is a distal femoral cemented modular prosthesis and is considered a viable option for post-bone tumor excision which allows early mobilization in a vulnerable subset of patients considering that it is a limb salvage procedure mostly [6]. Distal femur modular prosthesis is an implant, most commonly used for orthopedic surgery is used to replace the lower end (distal portion) of the femur which is damaged by fractures, bone tumors, or any other medical condition that affects the knee joint and lower part of the thigh bone [7].

While infections, tumor recurrence, or mechanical complications contribute towards early failures, implant fatigue or aseptic loosening contribute towards the majority of late failures. These lead to medical device failures, resulting in adverse events that can range from non-serious to serious and significantly impact the patient's quality of life, sometimes leading to severe health complications and prolonged recovery periods. For the past three decades, orthopedic oncologists have emphasized in favor of limb-sparing surgery for patients with sarcomas by gaining ever-expanding knowledge about the disease process along with advancement in imaging modalities, using adjuvant chemotherapies and better surgical techniques [8,9].

## Case presentation

**Figure 1:**
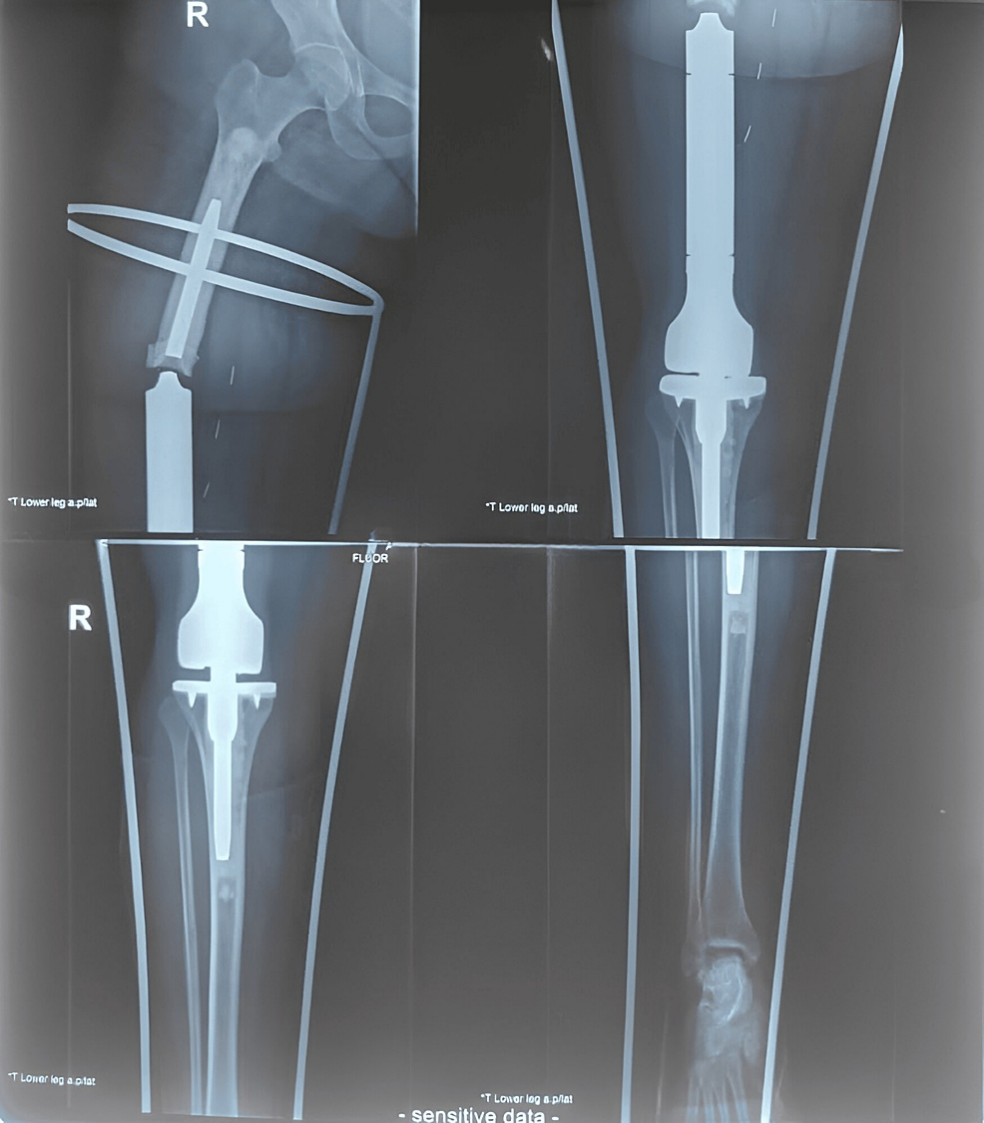
X-ray showing the broken implant of the megaprosthesis

A 24-year-old female patient reported to the pharmacology department in February 2024 complaining of excruciating pain in her right thigh since one week, along with an incapacity to bear weight on the right lower limb of the leg, which occurred after a seemingly innocuous act of turning in bed and with no history of significant trauma. The patient was experiencing this pain, sudden in onset, three years after the revision of the femoral stem and bush change of the megaprosthesis She was diagnosed with a broken implant (as shown in Figure 1), and on general examination, it was found that the patient was alert, conscious, and oriented.

Upon physical examination, it was discovered that there was no sinus or open wound but there was a healed scar measuring around 20 x 2 cm over the anteromedial portion of the distal thigh. On further investigation, it was found that the ankle and toes were moving and palpating. This was confirmed via inspection that there was no local rise in temperature, tenderness present over the mid and distal thigh, crepitus present over the mid-thigh region, abnormal mobility present over the mid-thigh anteroposterior and medical-lateral axis; distal pulses palpable. On the sixth day of her admission, a surgical procedure was carried out for the removal of the broken femoral component with its revision and reconstruction using cable wires and allogenic bone graft augmentation for femoral stem failure.

The patient's medical history revealed that she was previously diagnosed with osteosarcoma of the right distal femur in 2018. A surgical procedure was carried out in December 2018, for the excision of the tumor with megaprosthesis application, after which she was given four cycles of adjuvant chemotherapy (ACT). Further in 2020, she underwent a revision of the femoral stem and bush change of the megaprosthesis due to femoral stem failure.

The broken implant in this case resulted in the sudden onset of pain and the inability to bear weight on the leg. The sudden onset of these symptoms after turning in bed in a female patient describes the causal relationship between the medical device and the adverse reaction. Causality assessment of adverse reactions caused by medical devices can be established using the causality scale (https://www.ipc.gov.in/images/Guidance_Document_MvPI.pdf) as mentioned in the guidelines of the Materiovigilance Program of India (MvPI).

## Discussion

Osteosarcoma is found in the metaphysis of long bones, which are the proximal tibia, distal humerus, and distal femur, in descending order of occurrence. It is treated with a combination of surgery, adjuvant chemotherapy along radiotherapy. The radical excision of the tumor is a gold standard and is done after the spread of the tumor is assessed according to the Medical Devices Rule 2017 [10]. A cemented modular prosthesis is in the category of class-c medical devices (moderate-high risk), and over recent years it is believed that this cemented endoprosthesis fixation has become the gold standard [10,11].

Distal femur implant failure is a rare adverse event and according to Peivandi et al., these implant failures are caused due to the change in properties of metal, such as oxidation resistance, refinement of alloy, volume, configuration, and metal blends indicating poor quality of implants [12]. Another case study by Mwangi et al. describes the implant failures that occur due to biomedical material degradation resulting from mechanical issues like creep, wear, stress, and fractures, and physiochemical factors like biomolecular adsorption and biochemical reactions [13]. Other potential causes include design flaws, manufacturing contamination, varied sterilization methods, inadequate testing, packaging and shipping errors, and improper clinical handling or surgical techniques which contribute in causing implant failure [13].

The most profound complications of distal femur implant failure include fracture reoccurrences, infections, aseptic loosening as well as mechanical failure implants, surgical site infection, swelling, pain, loosening of implant components, dislocation of implant, cyst formation, and metalosis [14]. Despite improvements in the materials used for implants and in their design, mechanical failure and infection continue to limit the survival of endoprosthetic replacements (EPRs) largely depends upon the mechanical failures and infection while continuous improvements have been made over the years in the material science [7].

In our case report, a patient presented with pain in the right thigh, and incapacity to bear weight where it was found that the implant was broken. The common symptoms presented as pain in the thigh, difficulty in bearing weight, swelling, and an inability to walk or stand are generally associated with various medical conditions but these can also arise due to an implant malfunctioning or breakage. To ensure the reliability of the information, medical device adverse events that mimic pathological conditions need to be underscored. A significant rate of mechanical loosening has been reported for patients under the age of 60 rather than those above 60 while gender, diagnosis, and the duration of treatment have significantly no impact on the rate of complications or on survival of the prosthesis [7]. On comparing proximal humeral replacement, and proximal tibial along distal femoral replacements it was established that the longest mean time for failure was around 53 months [15]. Implants can trigger hypersensitivity reactions, leading to inflammatory responses and the formation of soft-tissue masses. These reactions can be formed by wear debris, particle toxicity, or an exaggerated immune response in the host [16].

The causality assessment was done on the basis of the scale issued by the National Coordination Centre-Materiovigilance Program of India (NCC-MvPI) where it was found that distal femur implant failure has a "probable" association (see Appendices) with the adverse event since the relationship between the investigational device and the adverse appears relevant, and cannot be fully explained by other factors [17]. However, further information may be necessary to fully understand the connection [17].

In India, the adverse event reporting associated with orthopedic implants has been minimal. Despite the existence of the MvPI for over seven years, aiming to monitor medical device safety, the data available shows that only a small percentage (2.4%) of reported adverse events are related to orthopedic implants. This is significantly lower compared to other medical devices. Our case report serves as crucial evidence to raise awareness among healthcare professionals and consumers. It not only encourages future reporting but also provides a valuable reference for future studies. Ultimately, this contribution will support the stabilization and effectiveness of the MvPI program. By highlighting real-world applications and outcomes, our report underscores the importance of vigilance and proactive measures in medical device safety. This emphasizes the importance of thorough medical assessments, including detailed examinations, imaging studies, and, when necessary, specific tests to determine whether symptoms stem from an implant malfunction or an underlying health issue. Prompt recognition and appropriate management of implant-related problems are vital to ensure patient safety and well-being.

## Conclusions

There is not enough evidence that lends credence to the idea that adverse events can also be related to medical devices. Thus, this case report about distal femur modular prosthesis implant failure is rare and can be considered as major evidence that could help not only healthcare professionals but also acknowledge the grave importance of materiovigilance. Over the recent few years, the idea of pharmacovigilance has been accepted globally and has also led to the identification of potential risks thereby implementing measures to mitigate them. The call for a global materiovigilance program is well-argued, aligning with the case's findings and broader implications.

## Figures and Tables

**Figure 2:**
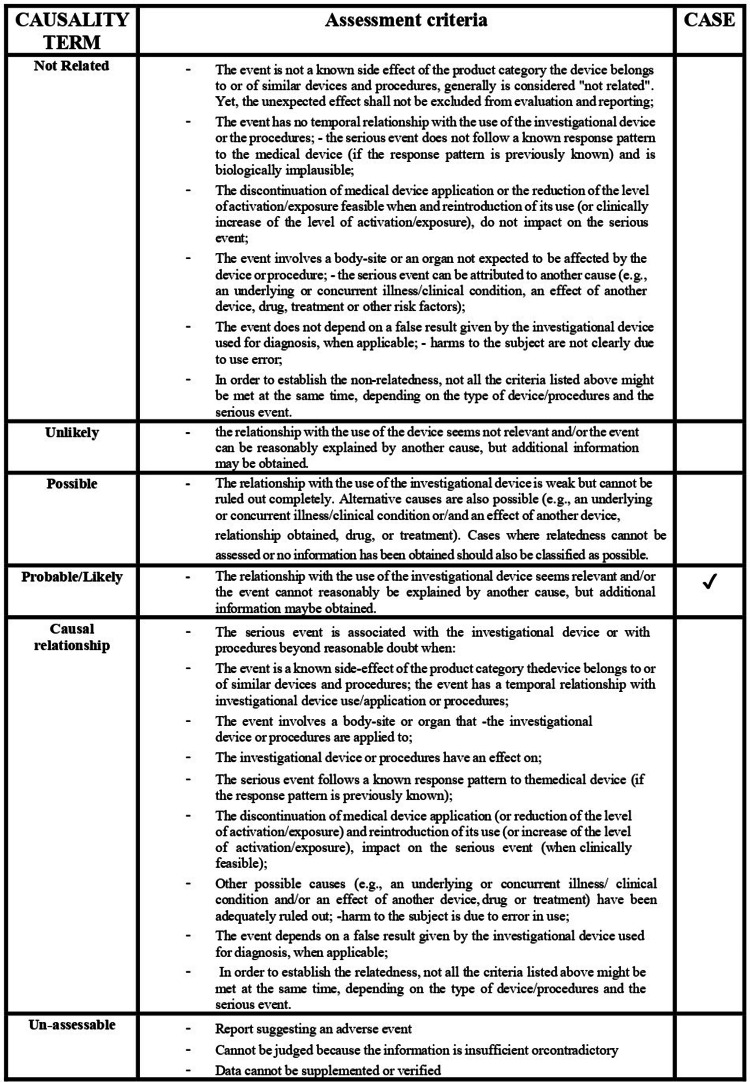
Causality assessment of medical device-related adverse events Source: [17]
